# Ensemble learning for prediction of the bioactivity capacity of herbal medicines from chromatographic fingerprints

**DOI:** 10.1186/1471-2105-16-S12-S4

**Published:** 2015-08-25

**Authors:** Hao Chen, Josiah Poon, Simon K Poon, Lizhi Cui, Kei Fan, Daniel Man-yuen Sze

**Affiliations:** 1 School of Information Technologies, University of Sydney, Sydney, Australia; 2 School of Medical Sciences & Health Innovations Research Institute (HIRi), RMIT University, Australia; 3 Key Laboratory of Advanced Control and Optimization for Chemical Processes, Ministry of Education, East China University of Science and Technology, Shanghai, China

**Keywords:** Chromatographic Fingerprints, Bioactivity Prediction, Ensemble Learning

## Abstract

**Background:**

Recent quality control of complex mixtures, including herbal medicines, is not limited to chemical chromatographic definition of one or two selected compounds; multivariate linear regression methods with dimension reduction or regularisation have been used to predict the bioactivity capacity from the chromatographic fingerprints of the herbal extracts. The challenge of this type of analysis requires a multi-dimensional approach at two levels: firstly each herb comprises complex mixtures of active and non-active chemical components; and secondly there are many factors relating to the growth, production, and processing of the herbal products. All these factors result in the significantly diverse concentrations of bioactive compounds in the herbal products. Therefore, it is imminent to have a predictive model with better generalisation that can accurately predict the bioactivity capacity of samples when only the chemical fingerprints data are available.

**Results:**

In this study, the algorithm of Stacking Multivariate Linear Regression (SMLR) and a few other commonly used chemometric approaches were evaluated. They were to predict the *Cluster of Differentiation 80 *(CD80) expression bioactivity of a commonly used herb, *Astragali Radix *(AR), from the corresponding chemical chromatographic fingerprints. SMLR provides a superior prediction accuracy in comparison with the other multivariate linear regression methods of PCR, PLSR, OPLS and EN in terms of *MSE_test _*and the goodness of prediction of test samples.

**Conclusions:**

SMLR is a better platform than some multivariate linear regression methods. The first advantage of SMLR is that it has better generalisation to predict the bioactivity capacity of herbal medicines from their chromatographic fingerprints. Future studies should aim to further improve the SMLR algorithm. The second advantage of SMLR is that single chemical compounds can be effectively identified as highly bioactive components which demands further CD80 bioactivity confirmation..

## Background

For thousands of years, herbal medicines have played a critical role in the primary health care systems for prevention and treatment of diseases, especially in Asian countries, such as China, India, Korea and Japan [1]. However, the therapeutic efficacies of herbal medicines are not evaluated or quantified due to the complexity of herbal extracts [2]. Herbal medicines contain complex compositions. A single herb may already include hundreds of natural constituents. In addition, synergistic or antagonistic interactive effects may exist among these compounds. Furthermore, the concentrations of active ingredients of a herb may vary substantially in different climates, cultivation conditions, storage conditions, extraction procedures, and other unknown factors [3]. This means that the bioactivity of herbal products for different batches may not be consistent, and this in turn may lead to unpredictable clinical therapeutic effects of these herbal products.

It is highly desirable to have a robust and efficient way to predict the bioactivity of herbal medicines. The use of whole chromatographic fingerprints is popular in solving herbal medicines' bioactivity prediction problems because the fingerprint provides a more comprehensive picture of herbal medicines [4]. A chromatographic fingerprint is a two-dimensional curve with time (min) as x-axis and intensity (mAU) as y-axis. There are two inherent characteristics of chromatographic fingerprints that should be noted. Firstly, as the intensity at every measuring time point in a chromatographic fingerprint is treated as the predictor variable, the number of predictor variables is normally larger than the number of samples investigated. This means that the intensity at every measuring time point will be treated as the predictor variable. For instance, a routine HPLC chromatographic fingerprint may contain about 10,000 time points within a 70 minutes' elution run constitute the high number of predictor variables in comparison with the limited number of the generally less than 100 grossly identified series of "peaks" in the chromatographic fingerprints.

Secondly, a chromatographic fingerprint may contain predictor variables that are not of high intensity due to different UV absorption rate, and contribute significantly to the overall bioactivity. Thirdly, various forms of multicollinearity may exist among predictor variables where some variables can be highly complementary.

A review of the literature shows that Principal Component Regression (PCR), Partial Least Squares Regression (PLSR), and variants of PLSR, such as Orthogonal Projections to Latent Structures (OPLS) and Elastic Net (EN) combined with PLSR (EN-PLSR), have been applied in prediction of biological activity from chromatographic fingerprints [2,4-12]. These approaches generate latent variables and measure the relationship between latent representations of the chromatographic fingerprints and bioactivities. Successful application of these algorithms may lead to useful prediction platforms for future bioactivity of unknown chromatographic fingerprints. However, if the number of latent variables to be used in the predictive model built by PCR, PLSR variants or EN, is not properly selected, over-fitting may occur and lead to poor generalisation performance of these models.

Based on a set of chromatographic fingerprints of *Astragali Radix (AR) *and their corresponding bioactivity of *Cluster of Differentiation 80 *(CD80) expression on the robust cell line representing human dendritic cells (DC) measured by flow cytometry, this study aims to propose an approach to develop predictive models with better and accurate generalisation ability to predict the bioactivity capacity of herbal medicines from their chromatographic fingerprints. Furthermore, the predictive accuracy of the model developed by the proposed approach will be evaluated in comparison to that of predictive models built by PCR, PLSR, OPLS and EN respectively.

This paper is organised as follows. The Methods section reviews the ensemble learning and describes the proposed method. In the Results and Discussion section, the performance of one real world data set is presented and discussed. The Conclusion section summarises the results of this study, and the Future Work section discusses potential tasks that are worthwhile for further investigation. It is then followed by a section of abbreviations used in this paper for easy reference.

## Methods

### Ensemble learning

It is a critical problem to machine learning when the training set is small or extremely small. The models built with a small training set may not be strong enough to accurately predict new instances. To improve the overall prediction accuracy, multiple models can be built on the training set, and prediction is made by incorporating and averaging over multiple models. This idea is called ensemble learning [13]. Ensemble learning combines a collection of base models (weak learners) to build a composite prediction model (strong learner). There are two main tasks of ensemble learning: constructing base models from the training data; and combining them to form a composite model [13]. Generally, there are three approaches to construct base models, also called ensembles.

**Data manipulation**. The first approach is to have a more effective use of the dataset by manipulation upon the data. By resampling the original training set according to a sampling distribution, such as bootstrapping, multiple training sets will be created. Then, a model can be created from each training-set using a learning algorithm [14]. Bagging [15] and boosting [16] are two ensemble methods which generate base models for further processing.

Bagging is a method that repeatedly samples from a data set according to bootstrap and train a base model on each bootstrap sample. The base models can be combined by simple averaging or majority voting. Bagging improves the prediction accuracy by reducing the variance of base models. However, the performance of bagging depends on the stability of base models [14].

Boosting is an iterative method that adaptively changes the distribution of training samples by assigning a different weight to each training sample. The adaptive weight assignment to the base models aims to direct the attention to the poorly classified or predicted training samples. Rather than averaging the results or taking majority voting from the ensembles, boosting combines the result from each base model according to its weight [14].

**Feature manipulation**. The second approach to construct base models is by to manipulate the input features, where each training set is formed by selecting a subset of input features. This approach works well when the data contain highly redundant features. The subset of features could be selected randomly or manually according to users' understanding of the data and domain experience. A typical ensemble approach in this category is Random Forests (RF) [14]. RF [17] is a combination of decision trees, in which each tree is generated according to the values of a random vector that is sampled independently and with the same distribution. It is claimed that RF is more robust to noise and runs faster [14].

**Multiple learning algorithms**. The third approach to construct base models is to use multiple learning algorithms. The aim is to obtain and to make advantage of the disagreement in sample classification or regression by using various learning algorithms. In other words, the different learning algorithms reveal the diversity of base models [18]. This approach is being adopted in this study to embrace the strengths of multiple algorithms.

Stacking [19] is an example of multiple learning algorithms. It aggregates the results from the base models, which are generated by different learning algorithms. Using a meta-learner, it tries to combine the results from these base learners in an optimal way to maximise the generalisation capability [20]. Breiman [21] proposed an approach called stacked regression. This approach was a linear combination of predictions from Subset Regression (SR) and Ridge Regression (RR). The researcher showed that the prediction performance of a stacked model was better than using SR or RR alone. Later on, Kedarisetti *et al*. [22] applied an improved stacking method, stacking C, to combine outputs from 5 classifiers, namely Logistic Regression (LR), Support Vector Machine (SVM), Nearest-neighbor algorithm and decision trees. They found that the classification performance using stacking C method outperformed those approaches using voting and multi-scheme.

It can be concluded from the above studies that stacking appears to be a good approach to combine predictions of several learning algorithms. The combined classifiers or regression models give better predictive accuracy than any single base learner. However, there is no unique way to combine the predictions of base learners using stacking. Simple linear regression with non-negative constraints is a simple way and it should usually work well [21].

### Stacking multivariate linear regression (SMLR)

Based on the approach of ensemble learning of stacking, in this study SMLR was proposed as the model for herbal medicines' bioactivity prediction. Essentially, SMLR combines the predictions from PCR [23], PLSR [24,25], OPLS [26] and EN [27,28] with non-negative constraints or coefficients. Then, the trained SMLR is used to predict the testing set of the chromatographic fingerprints.

The four underlying justifications for using PCR, PLSR, OPLS, and EN to build base learners are: firstly, these four methods can all work within the constraint of the size of sample is much smaller than the number of predictors. Secondly, PLSR and OPLS are the most commonly used methods to model the correlation between chromatographic fingerprints and the bioactivity of herbal medicines. Thirdly, PCR and EN are included to increase the diversity of base learners, as it has been reported that the biggest gains come when dissimilar sets of base learners are stacked [21]. PCR is different from PLSR and OPLS by extracting principal components that retain as much variation as possible in original space. The original data are projected to the new space (formed by the principal components), called scores. Then, PCR models the scores and the responses using Multivariate Linear Regression (MLR). EN is a regularisation method which adds two penalties to the least squares function (cost function) to penalise the regression coefficients of predictive models, this method is particularly useful when the dataset is sparse. Fourthly, only linear regression learning algorithms are considered in this study due to the fact that the linear models can be easily interpreted which is useful for the identification of highly bioactive regions or compounds in chromatographic fingerprints.

The outputs from the base learners are treated as inputs to train the meta-model, called Meta-Learning instances that are the predictions of base learners. The number of predictor variables of a Meta-Learning instance is the number of base learners. To train the meta-model, Meta-Learning training samples need to be formed. A 10-fold cross-validation is used for splitting an original training set into ten folds. Each time, one fold is held out to build the base learners using samples in the remaining nine folds. The base learners are trained on this dataset and to predict the responses in the holdout set. These predicted responses are then used to form the Meta-Learning training data. The rationale of these procedures is to ensure the Meta-Learning training data are able to reflect the true prediction performance of the base learners without bias and accurately [20].

The learning algorithm used for the development of the meta-model is RR [29]. It is under the constraint that the regression coefficients are non-negative, since the responses of a sample predicted using PCR, PLSR, OPLS, and EN models respectively are usually strongly correlated. RR adds *l*_2_ penalty to the least squares function (cost function) as a regularisation method. Constraining regression coefficients to be non-negative is to guarantee the response of a sample predicted by the meta-model will be within the range (minimum prediction of base learners, maximum prediction of base learners) in case the prediction of the meta-model is poor [21].

After determining the coefficients of PCR, PLSR, OPLS and EN models in the combination (meta-model), PCR, PLSR, OPLS, and EN models will be built using the original training set. When SMLR is used to predict a new sample; the sample is firstly predicted by PCR, PLSR, OPLS, and EN models that built on the original training set: each base model gives a predicted response of the sample. These predicted responses are then fed into the meta-model which combines them into a final predicted response.

The mathematical description of the strategy used for combining PCR, PLSR, OPLS, and EN in SMLR is as follows: suppose we have *k *base predictive models, $v_{1}\left(x\right),v_{2}\left(x\right),\ldots ,v_{k}\left(x\right)$, where *x *represents the predictor variables, stacking combines the predictions of these models rather than a single learner. The specific method for combination in SMLR is defined as follows: given an original training set $T=\{\left(y_{n},x_{n}\right),n=1,2,\ldots ,N$}, and a test sample $s=\left(y_{0},x_{0}\right)$, where *x_n _*is the predictor variable and each *x *is *p*-dimensional, *y_n _*is the response. Divide the data in *T *into 10 (almost) equal parts $T_{1},T_{2},\ldots T_{10}$ using tenfold cross-validation, and define $T^{j}=T-T_{j}$, where j=1,2,…,10. Using data in *T^j ^*train the PCR, PLSR, OPLS and EN models respectively, let $v_{k}^{j}\left(x\right)$, where k=1,2,3,4 denotes the predictive model built using *T^j^*. Specifically, $v_{1}^{j}\left(x\right)$ is the PCR model trained using *T^j^*, $v_{2}^{j}\left(x\right)$ is the PLSR model trained using *T^j^*, $v_{3}^{j}\left(x\right)$ is the OPLS model trained using *T^j^*, and $v_{4}^{j}\left(x\right)$ is the EN model trained using *T^j^*. Take the $\left\{\alpha_{k}\right\}$ to minimise:

(1) $$\underset{j}{\sum }\underset{\left(y_{n},x_{n}\right)\in T_{j}}{\sum }{\left(y_{n}-\alpha_{k}v_{k}^{j}\left(x_{n}\right)\right)}^{2}+\lambda \underset{k}{\sum }\alpha_{k}^{2}$$

under the constraint $\alpha_{k}\geq 0$. $\lambda \underset{k}{\sum }\alpha_{k}^{2}$ is *l*_2_penalty, which is used to penalise *α_k _*[21].

Then, develop $v_{k}\left(x_{n}\right)$ using the training set *T*. The response of the test sample *s *could be predicted as:

(2) $${ŷ}_{0}=\underset{k}{\sum }\alpha_{k}v_{k}\left(x_{0}\right)$$

Regression coefficients of original predictor variables need to be further calculated by using the following equation:

(3) $$\beta_{SMLR}=\alpha_{PCR}\times \beta_{PCR}+\alpha_{PLSR}\times \beta_{PLSR}+\alpha_{OPLS}\times \beta_{OPLS}+\alpha_{EN}\times \beta_{EN}$$

where $\beta_{SMLR}$, $\beta_{PCR}$, $\beta_{PLSR}$, $\beta_{OPLS}$, and$\beta_{EN}$ represent the regression coefficients of the original predictor variables in SMLR, PCR, PLSR, OPLS and EN models respectively. The $\alpha_{PCR},\alpha_{PLSR},\alpha_{OPLS},\alpha_{EN}$ are the coefficients of PCR, PLSR, OPLS and EN models respectively.

### Measures of predicting performance

In this study, mean-squared error (MSE) is used to measure the predictive performance of a predictive model. The calculation of MSE is shown as:

(4) $$MSE=\frac{\sum_{i=1}^{N}{\left({ŷ}_{i}-y_{i}\right)}^{2}}{N}$$

where ${ŷ}_{i}$ is the predicted response of the *i^th^*sample, *y_i _*is the observed response of the *i^th ^*sample, and *N *is number of samples.

The prediction error of a numeric prediction model on a test set is defined as:

(5) $$MSE_{test}=\frac{\sum_{i=1}^{T}{\left({ŷ}_{t}^{i}-y_{t}^{i}\right)}^{2}}{T}$$

where ${ŷ}_{t}^{i}$ is the predicted response of the *i^th ^*sample in the test set, $y_{t}^{i}$ is the observed response of the *i^th ^*sample in the test set, and *T *is the number of samples in the test set.

The prediction error of a numeric prediction model on a training set, also known as training error, is defined as:

(6) $$MSE_{training}=\frac{\sum_{i=1}^{P}{\left(({ŷ}_{p}^{i}-y_{p}^{i}\right)}^{2}}{P}$$

where ${ŷ}_{p}^{i}$ is the predicted response of the *i^th ^*sample in the training set, $y_{p}^{i}$ is the observed response of the *i^th ^*sample in the training set, and *P *is number of samples in the training set.

## Results and discussion

### Experiments

The experiment was designed to evaluate the performance of predictive models built by SMLR, and to compare the prediction performance of SMLR with that of PCR, PLSR, OPLS and EN respectively, using data of 72 chromatographic fingerprints of AR samples and their corresponding bioactivity, CD80.

The 72 chromatographic fingerprints of AR extracts were first pre-processed for baseline removal using adaptive Iteratively Reweighted Penalised Least Squares (airPLS) [30,31], alignment of chromatographic fingerprints using Correlation Optimized Warping (COW) [32-37], and standardisation. The chromatographic fingerprints after pre-treatment were then split into a training set and a test set using a bootstrap resampling procedure [20], and this procedure was repeated ten times, so there were ten training sets and ten test sets. PCR, PLSR, OPLS, EN and SMLR were applied to each training set to build prediction models, where they were evaluated using the corresponding test set.

### Data description

Based on the Traditional Chinese Medicine concepts, AR was chosen in this study because this herb has been believed to enhance the important "qi" of humans that elevates the general well-being and protection against diseases and infections. However, up to now, there is no quantifiable measurement of such "qi" in evidence-based medicine. The hypothesis taken in this study is that herbs that have been traditionally showing the desirable effect of "qi" enhancement will also upregulate the body's natural immunity - therefore the expression level of the costimulatory molecules of CD80 on our body's professional antigen presenting cells of DCs was measured [38-40].

Three batches of raw AR (AR-A, AR-B, AR-C) were used to prepare 72 extracts in total (24 extracts each) according to a modified extraction method based on the Chinese Pharmacopeia. These extracts were analysed by high performance liquid chromatography with diode array detector (HPLC-DAD) to obtain chromatographic fingerprints. Briefly, 4 grams of raw herb were pre-immersing with bi-distilled water (100, 150, 200 and 250 mL) for 12 hours and refluxed for 0, 1, 2, 3 and 4 hours. The mixtures were then filtered and concentrated under a rotary evaporator (Brand, Germany). AR extracts were finally obtained after lyophilisation. Each extract was stored under low humidity condition and was kept for biological assay within 3 months. All the extracts before chromatographic analysis and biological assay were filtered under 0.2um filter. Bi-distilled water was produced in-house by Milli-Q^® ^Advantage A10 water purification systems (Millipore; USA) and filtered with 0.22 μm Millipak^®^. All other chemicals and reagents used were of analytical grade unless indicated otherwise. The bioactivity CD80 was measured through a THP-1 DC flow cytometric platform.

Since a chromatographic run was 70 min long and the sampling rate was 0.0067 min, the number of points acquired per chromatographic fingerprint of AR was 10,501. In other words, each chromatographic fingerprint is a signal graph with 10,501 data points, with the interval between two points being 0.0067 min.

Digitised chromatographic fingerprints of AR were gathered in a 72 by 10,501 matrix *X *where each row represents the fingerprint of one sample and the 10,501 variables (measuring time points) are the columns. The corresponding bioactivity capacities were represented as a 72-row vector *y*. When building predictive models, all these 10,501 points of each digitised chromatogram were used as predictor variables, and the CD80 capacity of each sample is the response.

## Results

The *MSE_test _*and *MSE_training _*of each model built by PCR, PLSR, OPLS, EN and SMLR are listed in Table 1 and 2. The numbers in the first column of Table 1 and 2 represent the predictive model built using the first training set, second training set, ... etc.

**Table 1:** the *MSE_test _*of each predictive model build by PCR, PLSR, OPLS, EN and SMLR

*MSE_test _*					
**Test Set**	**PCR**	**PLSR**	**OPLS**	**EN**	**SMLR**

1	0.18545	0.17693	0.17693	0.14299	0.16524
2	0.26334	0.29223	0.29223	0.27688	0.27026
3	0.22515	0.21094	0.21094	0.18428	0.18602
4	0.27171	0.25480	0.25480	0.26347	0.25632
5	0.20965	0.19050	0.19050	0.22645	0.19704
6	0.33327	0.32225	0.32225	0.30530	0.30847
7	0.23789	0.22115	0.22115	0.24703	0.21343
8	0.25015	0.22506	0.23109	0.24708	0.22395
9	0.18473	0.17900	0.17900	0.20893	0.18507
10	0.27251	0.26961	0.26961	0.27560	0.26843
**Mean**	**0.24339**	**0.23425**	**0.23485**	**0.23780**	**0.22742**

**Table 2:** the *MSE_training _*of each predictive model build by PCR, PLSR, OPLS, EN and SMLR

*MSE_training_*					
**Training Set**	**PCR**	**PLSR**	**OPLS**	**EN**	**SMLR**

1	0.05722	0.01562	0.01562	0.04591	0.02273
2	0.07726	0.02304	0.02304	0.0688	0.0339
3	0.05432	0.00992	0.00992	0.00278	0.01183
4	0.0372	0.02113	0.02113	0.17149	0.03334
5	0.02094	0.01609	0.01609	0.05693	0.0168
6	0.04337	0.02291	0.02291	0.20607	0.03839
7	0.03314	0.02129	0.02129	0.04031	0.01823
8	0.04345	0.01429	0.02959	0.04318	0.02377
9	0.0417	0.03122	0.03122	0.02044	0.02484
10	0.07736	0.01078	0.01078	0.07534	0.02725
**Mean**	**0.0486**	**0.01863**	**0.02016**	**0.07313**	**0.02511**

The difference of the predicted bioactivity capacity of a test sample and its corresponding observed bioactivity capacity is another measurement to study and to analyse. The criteria of how good the bioactivity capacity of a new sample is predicted are listed in Table 3.

**Table 3:** Criteria of goodness of prediction

Differences (%)	How Good is the Prediction
≤ 10	Excellent
>10 & ≤20	Good
>20 & ≤30	Acceptable
> 30	Poor

The difference between the predicted CD80 expression and the observed response of a test sample can be divided into different grades. In general, CD80 flow cytometric expression for replicates of the same RA extract have a standard deviation of about 2% of the mean value. Thus a prediction of <10% difference can be considered as excellent; and between 10-20% is good and so on. The first column of Table 3 is the difference of the predicted bioactivity capacity of a test sample and its corresponding observed bioactivity capacity in percentage. The second column displays the four levels, namely excellent, good, acceptable and poor of how good the prediction is.

The average difference (in percentage) of the predicted bioactivity capacity of test samples and their corresponding observed bioactivity capacity in each test set (1,2, ..., 10) for PCR, PLSR, OPLS, EN and SMLR models can be found in Table 4. The numbers in the first column of this table are the same as Table 1 and 2. In order to analyse whether the mean *MSE_test _*and the mean differences of predicted responses and observed responses of PCR, PLSR, OPLS, EN and SMLR are significantly different, one-way ANOVA was applied. The results are shown in Table 5 and 6.

**Table 4:** Average difference of predicted responses and observed responses in each test set for each model

Difference (%)					
**Test Set**	**PCR**	**PLSR**	**OPLS**	**EN**	**SMLR**

1	12.72	12.089	12.089	10.9955	11.972
2	14.6231	14.972	14.972	13.635	14.2868
3	12.8561	12.6299	12.6299	11.5313	12.1758
4	15.1355	14.5171	14.5171	15.3884	14.8151
5	13.0635	12.3538	12.3538	13.8607	12.8326
6	14.6371	13.954	13.954	13.9972	13.9497
7	15.2913	14.5146	14.5146	14.8474	14.132
8	14.4245	14.0604	14.3823	13.9682	13.9149
9	12.1609	11.7033	11.7033	12.918	12.0041
10	14.4008	13.6742	13.6742	14.2981	13.9341
**Mean**	**13.9313**	**13.4468**	**13.479**	**13.544**	**13.4017**

**Table 5:** One-way ANOVA for the *MSE_test_* of each model of PCR, PLSR, OPLS, EN and SMLR

*Source of Variation*	*SS*	*df*	*MS*	*F*	*P-value*	*F crit*
**Between Groups**	0.00135	4	0.00034	0.14765	**0.96311**	2.57874
**Within Groups**	0.10264	45	0.00228			
**Total**	0.10399	49				

**Table 6:** One-way ANOVA for differences of predicted and observed responses of PCR, PLSR, OPLS, EN, SMLR

*Source of Variation*	*SS*	*df*	*MS*	*F*	*P-value*	*F crit*
**Between Groups**	0.00018	4	4.56E-05	0.32640	**0.85878**	2.57874
**Within Groups**	0.00629	45	0.00014			
**Total**	0.00647	49				

## Discussion

Comparing the *MSE_test _*of predictive models built by PCR, PLSR, OPLS, EN and SMLR, SMLR had the smallest mean (0.22742), followed by PLSR (0.23425), OPLS (0.23485), EN (0.23780) and PCR (0.24339), though the differences of them were small, only at the second, third, or even fourth decimal place. And these differences were not statistically significant according to the one-way ANOVA analysis results (*F *(4,45) = 0.15*, p = *0.96).

In terms of the average difference of predicted responses and observed responses in each test set for models built by PCR, PLSR, OPLS, EN and SMLR, SMLR had the smallest mean differences which is 13.40171%. PLS came second (13.44682%), OPLS came third (13.47901%), followed by EN (13.54398%), and PCR (13.93128%). Again, the differences among the models were also small and not statistically significant; the one-way ANOVA result is *F *(4,45) = 0.33, *p *= 0.86.

The mean differences between predicted CD80 capacity and observed CD80 capacity for SMLR, PCR, PLSR, OPLS and EN are around 13% to 14%, which means their predictions for CD80 capacity from chromatographic fingerprints of AR samples are good, according to the goodness criteria of predicting the bioactivity capacity of a sample (Table 3).

The predicting performances of PCR, PLSR, OPLS, EN and SMLR models, in terms of *MSE_test _*and differences between predicted responses and observed responses of test samples, had the following ranking: SMLR, PLSR, OPLS, EN and PCR. However, the improvement of predictive accuracy was not significant.

SMLR did not significantly improve the predictive accuracy. This may be because the learning algorithm applied in SMLR did not combine the predictions of base learners in the optimal way. Another reason could be that the size of samples is too small. There are not enough samples to train the meta-model. In addition, if more learning algorithms were used to create base models, the predictive accuracy might be improved to some extent.

SMLR could also be applied in other domains, not just for measuring the relationship between chromatographic fingerprints and the bioactivity capacity of herbal medicines. It is designed to solve the prediction problem when the number of samples is far less than the number of predictor variables, and when the predictor variables are highly correlated.

## Conclusions

In this article, SMLR was presented as an algorithm that could develop predictive models for predicting bioactivity capacity of herbal medicines from their chromatographic fingerprints. SMLR is a meta-learner that works on the results the constituent base-learners. Its generalization capability becomes more obvious when its constituent base-learners are more diversified in nature. The prediction performances of models built by using SMLR for predicting CD80 from the chromatographic fingerprints of AR samples are superior to PCR, PLSR, OPLS, and EN in terms of *MSE_test _*and differences between predicted responses and observed responses of test samples. However, the differences among the models are small and not statistically significant.

## Future work

Future studies should aim to further improve the predictive accuracy of the SMLR algorithm. For instance, more learning algorithms can be attempted to generate more training instances for the Meta-Learner. Another possible investigation is the identification of base learners that has maximum diversity, so that the Meta-Learner can have optimal performance owing to the complementary behavior of the constituent base learners. In addition, other linear or non-linear learning algorithms could be designed and applied for investigating how to combine the output of the base learners in order to improve the predictive accuracy. Furthermore, it is important to perform CD80 bioactivity testing on those single chemical compounds that has been predicted to be the highly bioactive components. This may then demonstrate that this chemometrics prediction platform has a strong potential to be an effective drug discovery platform as well.

In this study, SMLR has been evaluated using only one dataset, which does not appear to be that sufficient. In the future, a few other datasets, with both quality chemical fingerprints and corresponding bioactivity results, should be used for evaluation of the proposed approach if possible. It is envisaged that this same platform may be applicable for the analysis of other kinds of mixture datasets not limited to the chromatographic fingerprints and bioactivity capacity.

## List of abbreviations used

**airPLS: **adaptive Iteratively Reweighted Penalized Least Squares; **ANOVA: **Analysis of Variance; **AR: ***Astragali Radix; ***CD80: ***Cluster of Differentiation 80; ***COW: **Correlation Optimized Warping; **DC: **Dendritic cell; **EN: **Elastic Net; **EN-PLSR: **Elastic Net combined with PLSR; **HPLC-DAD: **High Performance Liquid Chromatography with Diode array detector; **LR: **Logistic Regression; **MLR: **Multivariate Linear Regression; **MSE**: Mean-Squared Error; **OPLS: **Orthogonal Projections to Latent Structures; **PCR: **Principal Component Regression; **PLSR: **Partial Least Squares Regression; **RF: **Random Forests; **RR: **Ridge Regression; **SMLR: **Staking Multivariate Linear Regression; **SR: **Subset Regression; **SVM: **Support Vector Machine.

## Competing interests

The authors declare that they have no competing interests.

## Authors' contributions

HC designed and implemented the SMLR algorithm, conducted the experiment, analysed the results, and drafted the manuscript. JP and SKP supervised the manuscript, conceived of the whole method and revised the manuscript. LC gave suggestions on developing the SMLR algorithm. KF provided domain knowledge of Traditional Chinese Medicine and critical discussion of the approach to this study. DS provided domain knowledge of Traditional Chinese Medicine, the data used in the experiment and reviewed the manuscript.

## Declarations

There is no grant agency to support the publication of this paper. The publication fee is sourced from the research account of Josiah Poon in the School of IT, University of Sydney.

This article has been published as part of *BMC Bioinformatics *Volume 16 Supplement 12, 2015: Selected articles from the IEE International Conference on Bioinformatics and Biomedicine (BIBM 2014): Bioinformatics. The full contents of the supplement are available online at http://www.biomedcentral.com/bmcbioinformatics/supplements/16/S12.
